# The Oligomycin-Sensitivity Conferring Protein of Mitochondrial ATP Synthase: Emerging New Roles in Mitochondrial Pathophysiology

**DOI:** 10.3390/ijms15057513

**Published:** 2014-04-30

**Authors:** Manuela Antoniel, Valentina Giorgio, Federico Fogolari, Gary D. Glick, Paolo Bernardi, Giovanna Lippe

**Affiliations:** 1 Department of Biomedical Sciences, University of Padova, via Ugo Bassi 58/B, 35121 Padua, Italy; E-Mails: manuela.antoniel@studenti.unipd.it (M.A.); vgiorgio@bio.unipd.it (V.G.); bernardi@bio.unipd.it (P.B.); 2 Department of Food Science, University of Udine, via Sondrio 2/A, 33100 Udine, Italy; 3 Department of Biomedical Sciences, University of Udine, p.le Kolbe, 33100 Udine, Italy; E-Mail: federico.fogolari@uniud.it; 4 Department of Chemistry, Graduate Program in Immunology, University of Michigan, Ann Arbor, MI 48109, USA; E-Mail: gglick@umich.edu

**Keywords:** mitochondria, oligomycin-sensitivity conferring protein (OSCP), cyclophilin D (CyPD), F_O_F_1_ ATP synthase dimer, permeability transition pore (PTP)

## Abstract

The oligomycin-sensitivity conferring protein (OSCP) of the mitochondrial F_O_F_1_ ATP synthase has long been recognized to be essential for the coupling of proton transport to ATP synthesis. Located on top of the catalytic F_1_ sector, it makes stable contacts with both F_1_ and the peripheral stalk, ensuring the structural and functional coupling between F_O_ and F_1_, which is disrupted by the antibiotic, oligomycin. Recent data have established that OSCP is the binding target of cyclophilin (CyP) D, a well-characterized inducer of the mitochondrial permeability transition pore (PTP), whose opening can precipitate cell death. CyPD binding affects ATP synthase activity, and most importantly, it decreases the threshold matrix Ca^2+^ required for PTP opening, in striking analogy with benzodiazepine 423, an apoptosis-inducing agent that also binds OSCP. These findings are consistent with the demonstration that dimers of ATP synthase generate Ca^2+^-dependent currents with features indistinguishable from those of the PTP and suggest that ATP synthase is directly involved in PTP formation, although the underlying mechanism remains to be established. In this scenario, OSCP appears to play a fundamental role, sensing the signal(s) that switches the enzyme of life in a channel able to precipitate cell death.

## 1. Introduction

Mitochondria are cytoplasmic double-membrane-delimited organelles of variable size, with a diameter between 0.5 and 5 μm, which are organized in a dynamic network [1] and are responsible of the aerobic production of ATP by the oxidative phosphorylation system (OXPHOS). In addition, mitochondria participate in intermediary metabolism and are involved in the early stages of cell death, where the Ca^2+^-dependent permeability transition pore (PTP) plays a key role [2]. OXPHOS catalyzes the oxidation of fuel molecules and the concomitant synthesis of ATP via five complexes located in the inner mitochondrial membrane (IMM), which is highly folded into cristae. Briefly, Complexes I, III and IV of the respiratory chain catalyze a sequential series of redox reactions, resulting in the reduction of oxygen to water; at the same time, they pump protons across the IMM into the intermembrane space (IMS), generating a proton electrochemical gradient that provides the driving force for the synthesis of ATP from ADP and inorganic phosphate (Pi) within Complex V (or F_O_F_1_ ATP synthase).

Intriguingly, recent data suggest that Complex V has a second function, as it is able to generate Ca^2+^-dependent currents with features indistinguishable from those of the PTP, as assessed after isolation in the form of dimers, which represent its physiological state in the IMM [3]. The enzyme of life appears therefore to be directly involved in PTP formation to stimulate cell death [4]. The OSCP (oligomycin-sensitivity conferring protein) subunit of ATP synthase appears to play a unique role, being the site of interaction of cyclophilin (CyP) D, a matrix protein that favors PTP opening, as will be discussed below. CyPD is also the downstream effector of kinase signaling pathways, which target the PTP, such as hexokinase II and the Akt-ERK-GSK3 axis, whose dysregulation is a hallmark of several neoplastic cell models [5]. Post-translational modifications (PTMs) of OSCP have also been reported [6–8], but their functional implications are still to be defined. These observations suggest that CyPD-OSCP interaction mediates a survival or death signal modulating the PTP closure/opening, although the underlying mechanism remains to be establish.

The IMM also contains many transporters or carriers that translocate nucleotides, inorganic ions and metabolites, including phosphate, thereby determining the compartmentalization of the metabolic functions of mitochondria. The mitochondrial proteome ranges between about 800 (yeast) and 1500 (human) different proteins [9], with relevant differences between organs [10]. Although the majority of mitochondrial proteins is encoded in the nucleus and post-translationally imported into the organelle by a complex protein-import machinery [11], some subunits of the OXPHOS (13 in humans) are encoded by the mitochondrial genome (mtDNA), which is present in several copies per organelle and is exclusively maternally inherited.

Defects in mitochondrial DNA (mtDNA) have been associated with mitochondrial diseases, which encompass a wide variety of degenerative diseases and may also play a role in aging and cancer [12]. Complex V deficiency due to mutations in the mtDNA-encoded subunits a and A6L is a very rare OXPHOS deficiency. Phenotypic variations in disease severity depend mainly on the fraction of mutated mtDNA percent (*i.e.*, the level of heteroplasmy), as observed in Maternally inherited Leigh syndrome (MILS) and neuropathy, ataxia and retinis pigmentosa (NARP) syndrome, caused by subunit a (*MT-ATP6*) mutations. Mitochondrial diseases due to genetic defects in the nuclear genes affecting one structural subunit, one assembly and one ancillary factor have also been described [13]. Deficiency in ATP synthesis appears to be the main pathogenic factor, although other mechanisms are involved, such as the generation of reactive oxygen species (ROS) [14–16]. On the other hand, the novel function of ATP synthase in PTP formation makes this picture much more complex, considering the role played by PTP and by its dysregulation in a variety of diseases characterized by altered cell death, which include ischemia-reperfusion injury of the heart and brain, muscular dystrophies, neurodegeneration and cancer [5,17].

## 2. ATP Synthase Structure

The mitochondrial ATP synthase is a 600-kDa multisubunit complex. Its molecular structure and catalytic mechanism were understood by the seminal work of the Nobel Laureates, Mitchell, Boyer and Walker [18–20], which revealed its complexity and the functional steps driving the synthesis of ATP. In addition to the IMM, ATP synthase is present in the thylakoid membrane of chloroplasts, and in the plasma membrane of bacteria and mammalian cells, although with opposite orientation [21,22]. It is well conserved, considering its structural complexity and the early divergence of bacteria, plants and animals [23]. Proton translocation and synthesis of ATP from ADP and Pi are coupled by a unique mechanism: subunit rotation. The electrochemical energy contained in the proton gradient is converted into mechanical energy in the form of subunit rotation and back into chemical energy as ATP [24–27]. The enzyme is also able to work in the direction of ATP hydrolysis, sustaining the formation of the proton gradient, when there is a loss of membrane potential. In both directions, Mg^2+^ is essential for catalysis, and it can be replaced by other divalent cations, such as Mn^2+^ and Co^2+^. Intriguingly, Ca^2+^ ions, which induce PTP formation, only sustain ATP hydrolysis by F_1_, which is not coupled to proton translocation both in bacteria [28] and in mammals [29].

Traditionally, ATP synthase is divided into two subcomplexes, the membrane-embedded F_O_ subcomplex, through which the protons flow, and the soluble F_1_ subcomplex. The latter is always composed of three copies of each of subunits α and β (which carry the nucleotide binding sites) and one each of subunits γ, δ and ɛ (which constitute the central stalk of the complex). Subunit γ rotation within α_3_β_3_ takes each of the three β subunits through the conformations, β_DP_, β_TP_ and β_E_, thereby synthesizing three Mg^2+^-ATP molecules during each 360° rotation [30].

The inhibitor protein, IF_1_, binds to F_1_, at a α/β interface, under energy deficiency [31,32], *i.e.*, at low pH and membrane potential, when the enzyme hydrolyzes rather than synthesizes ATP. Therefore, IF_1_, which is evolutionarily conserved from yeast to mammals, is considered responsible for the beneficial (at least partial) inhibition of F_O_F_1_ during ischemia both in *in vitro* experimental models, as well as *in vivo* [33], as also demonstrated by our group in anaesthetized open-chest goat heart [34]. Unexpectedly, IF_1_-knockout mice grew without defects, but their behavior against stressful situations have not been tested [35].

**Figure 1.**
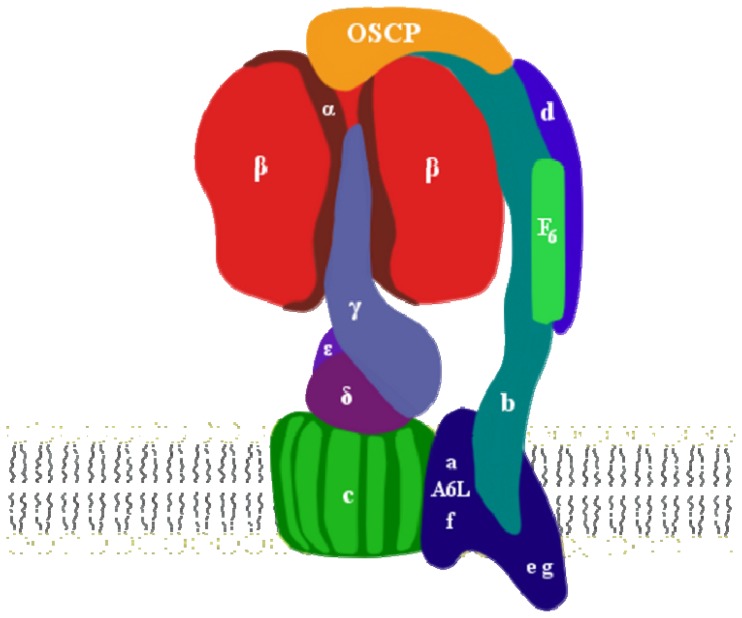
Schematic representation of F_O_F_1_ ATP synthase as a monomer.

In the simplest form of the enzyme, such as that of bacteria, like *Escherichia coli*, F_O_ consists of three different subunits, a, b and c, with stoichiometry ab_2_c_9–12_ [23]. Subunits a and c form the proton channel, while subunit b links the proton channel to F_1_. The animal mitochondrial F_O_ sector has a more complicated structure, since ten subunits have been identified, a, b, c, F_6_, d, e, A6L, f, g and OSCP [36]. The a, b and c subunits share great similarity with the bacterial subunits, except for subunit c stoichiometry which comprises only eight copies, thus reducing the bioenergetic cost of ATP synthesis [37], whereas the remaining subunits are unique to the mitochondrial complex (Table 1). The membrane-embedded part of F_O_ comprising subunits e, f, g and A6L is connected to F_1_ by a complex peripheral stalk, composed by one copy each of subunit b, d, F_6_ and OSCP, which is important to prevent the co-rotation of α and β subunits with γ and, therefore, the loss of enzyme efficiency. OSCP is a 213-amino acid protein (including the 23 amino acid mitochondrial leader sequence) located on the top of F_1_ [38], which is analogous to *E. coli* δ-subunit and is highly conserved among mammals [39]. Besides these subunits, in beef, rat and man, two hydrophobic proteins, namely a 6.8-kDa proteolipid (called MLQ) [40,41], and α1 acid glycoprotein/diabetes-associated protein in insulin-sensitive tissue (called AGP/DAPIT) [42], are associated with the F_O._ All together, the mitochondrial membrane domain is constituted by approximately 30 trans-membrane α-helices [43] (Figure 1).

ATP synthase is commonly isolated as a functional monomer, but this is not the physiological state in yeast, mammalian and plant mitochondria [44,45]. Biochemical and electron microscopy studies demonstrated that within the IMM, the enzyme is organized in dimers associated with forming long rows of oligomers located at the cristae ridges to maintain a high local curvature [3] and normal cristae morphology [46,47]. Dimers interact within the IMM through the F_O_ subunits [48] with the peripheral stalks turned away from each other [38]. The proposed roles of the ATP synthase oligomers are higher efficiency and higher stability [3,49]. Consistently, in aged mitochondria of *Podospora anserina*, the disappearance of the typical IMM architecture and impairment of ATP synthesis were concomitant to ATP synthase dimer dissociation and formation of IMM vesicles [50]. Intriguingly, electron tomography evidenced the binding of a bell-shaped protein, possibly CyPD, between the F_O_-F_O_ of dimers at the bases of the peripheral stalks in early-vesicular mitochondria followed by its dissociation from monomers in aged mitochondria. These observation prompted the authors to propose a role of CyPD in promoting dimer dissociation. We found that CyPD interacts with OSCP in the ATP synthase dimers promoting PTP formation [2,4], as will be discussed below. If CyPD binding between the two F_O_ will be confirmed by further investigation, the existence of distinct CyPD binding sites on ATP synthase dimers may be proposed.

The structural properties of dimers were initially characterized in yeast, where genetic approaches, cross-linking analyses and electron microscopy images established preferential interactions within the IMM, mainly through the Su6 [51], Su4 [52], e [53] and g subunits [54], which formed Su6/Su6, Su4/Su4 and e/g interactions, and also through the yeast-specific F_O_ subunits, h and i [55]. In keeping with its general occurrence, the involvement of subunits a [56], e [57] and g [44] in ATP synthase dimerization has been demonstrated also in mammals. In addition, a second interface (named the oligomerization interface) has been described in yeast; this interface was stabilized through e/e and g/g interactions allowing oligomer formation [45]. However, the existence of an oligomerization interface has been criticized based on electron cryotomography data of mitochondria from different sources, including bovine heart, which evidenced variable distance between dimers, making direct protein contacts difficult [3]. Moreover, electron cryotomography showed that dimers of all species display fixed angles between two F_1_-F_1_ with an angle >70° [3], in contrast with previous single-particle electron microscopy images, which indicated angles ranging from 40° [58,59] to 70°–90° [48,60]. It has been proposed that the 40° dimers form upon the inhibitory binding of IF_1_ [3], which was contained in considerable amounts in isolated dimers [58]. However, although IF_1_ was not essential for ATP synthase dimerization both in yeast [61] and mammals [62], we found that in cardiomyoblasts undergoing cardiac differentiation, IF_1_ binding promoted dimer/oligomer stabilization, possibly through a non-inhibitory interaction, which remains to be clarified. In fact, in differentiated cells, an increased IF_1_ binding occurred in parallel with an increase of ATP synthesis, as already reported in human HeLa cells [63] overexpressing IF_1_, as well as of ATP hydrolysis [64]. Intriguingly, a pH-independent interaction of IF_1_ with OSCP has been described [65], whose functional implication remains to be established. A stabilizing effect on bovine ATP synthase dimers has been also reported for the matrix metalloprotein, called factor B, which interacts with the e and g subunits of F_O_ [66].

## 3. OSCP: Location and Structure

It has long been established that OSCP is essential for conferring sensitivity to the inhibition of ATP synthase by the antibiotic, oligomycin [67], although oligomycin does not bind OSCP. Sensitivity to oligomycin can be defined in two contexts. The first relates to the “coupling” of the enzyme assayed as the oligomycin-sensitive ATP hydrolytic (ATPase) activity. It is a measure of the structural integrity of the enzyme, since detachment of F_1_ from F_O_ leads to a decrease in oligomycin-sensitive ATPase activity [68–72]. Under these conditions, ATP hydrolysis by F_1_ still takes place, but ATP hydrolysis is no longer coupled to proton translocation, and the sensitivity to oligomycin is lost, demonstrating that oligomycin inhibits proton transport in F_O_, thus affecting the *V*_max_ of ATP hydrolysis, only when F_O_ is correctly bound to F_1_, *i.e.*, in the coupled enzyme [70]. In this context, depletion/reconstitution studies in isolated membranes demonstrated that OSCP is absolutely necessary in coupling proton translocation to ATP synthesis [71]. In keeping with this key function of the subunit, yeast OSCP knockouts failed to survive under normal growth conditions [73].

The second context relates to the ability of the assembled ATP synthase complex to bind oligomycin. In yeast, the oligomycin resistance phenotype is conferred by mutations in one of the two mitochondrially encoded subunits, a (Su6) and c (Su9), indicating that the oligomycin-binding site encompasses the *C*-terminal region of Su6 and the transmembrane domains of Su9 [74]. This is consistent with the role of these subunits in proton translocation through F_O_, which requires the concerted action of both.

Boyle and Colleagues have examined the effect of a single residue change at a conserved glycine (Position 166) of yeast OSCP [75]. A G166N mutant allowed enzyme assembly into functional complexes and was able to grow on ethanol, albeit with a slower generation time. Mutant cells demonstrated a relative insensitivity to oligomycin during State 3 respiration, as well as a passive leak of protons across the IMM. Moreover, ATP synthase complexes containing the G166N mutation were found to be unstable, being more susceptible to dissociation than complexes containing native OSCP. These properties suggested that altered protein-protein interactions between OSCP and the α_3_β_3_ hexamer partially uncoupled the enzyme. Interestingly, binding of 17β-estradiol to OSCP promoted an intrinsically uncoupled state of ATP synthase, while the enzyme was actually catalyzing the ATP synthesis in rat liver mitochondria [76]. Fluorescence resonance energy transfer (FRET) measurements have documented the stress developing between F_1_ and OSCP during ATP hydrolysis, which appears to match its role as part of the peripheral stalk [77].

Intriguingly, OSCP binds isolated F_1_ in bovine, but not yeast, mitochondria [70,78] Moreover, *in organello* pulse-labeling and pulse-chase experiments run in yeast seem to indicate that OSCP binding is the last step in the assembly of F_O_F_1_ and that it occurs independently of the two pathways that converge to form the whole ATP synthase from the F_1_/Su9-ring and Su6/Su8/peripheral stalk subcomplexes [79]. Consistent with these data, reducing OSCP levels in human cells did not alter the expression of other F_O_F_1_ subunits, as well as of the respiratory chain complexes [4,78]. Interestingly, in a model of Leigh syndrome obtained in a conditional knockout mouse heart for the mtDNA regulator, *Lrpprc* (leucine-rich pentatricopeptide repeat containing protein), the appearance has been recently observed of ATP synthase subcomplexes lacking OSCP and A6L. These subcomplexes contained a high amount of IF1, which blocked the ATP hydrolysis at physiological pH, counteracting the overall impairment of ATP production [16].

Rees and Colleagues [80] established the physical location of OSCP in the peripheral stalk by analyzing the crystal of a F_1_-peripheral stalk subcomplex and demonstrated that the subunit sits on top of the F_1_ domain (see Figure 1), in agreement with earlier experiments [81]. Its *N*-terminal domain (Residues 1–113) contains six α-helices and shows a similar fold to that of the *N*-terminal fragment of recombinant OSCP analyzed by NMR [82]. Helices 1 and 5 provide the binding site: (i) for Residues 6–17 of subunit α_E_ (which contacts β_E_ in α_3_β_3_ subcomplex), largely via hydrophobic interactions and two possible charge-charge interactions through R15 and E91; and (ii) for E7 and R94 of subunits α and OSCP, respectively [80]. These interactions are similar to those determined with isolated OSCP and synthetic peptides from the *N*-terminals of mitochondrial and bacterial α subunits [81,83]. There is another region of interaction involving Residues 1–14 of the OSCP with the F_1_ *N*-terminal β-barrel domains of the β_DP_-subunit [80].

The structure of the *C*-terminal domain of OSCP, which is unstructured in the isolated protein [82], consists of a β-hairpin, which precisely locates along the α/β interface [38], followed by two α-helices, H7 and H8 [80]. H8 of the OSCP forms a three-helix bundle with the *N*-terminal α-helix of F_6_ and a segment of subunit b. The *C*-terminal of subunit b also packs against helix H7 of the OSCP, resulting in an extensive interface between OSCP and subunits b and F_6_.

## 4. Post Translational Modifications of OSCP Subunit

Information on post-translational modifications (PTM) of ATP synthase subunits has increased with the development of new techniques, such as PTM-specific mass spectrometry (MS)-based methods, selective dyes (ProQ Diamond) and specific antibodies. The association of several of these PTMs with specific biological processes, *i.e.*, PDGF-mediated phosphorylation of the δ subunit [84,85], or diseases (such as type 2 diabetes [86]) has been found. The challenge is now to find direct functional implications for these PTMs. The OSCP subunit has been reported to be glycosylated [7], phosphorylated [6,87–90] and acetylated [8,91] (Table 2).

Although the glycosylation of secreted, membrane and nucleocytosolic proteins has been well studied, information on the glycosylation of mitochondrial proteins is limited [93,94]. Recently, different authors have proposed that several proteins with mitochondrial function or with mitochondrial-annotation and, thus, putative mitochondrial function may be glycosylated [95–98].

Berninsone and Burnham-Marusich [7] identified glycosylated isoforms of OSCP and the d subunit in bovine heart mitochondria by using a leptin resin. The same authors also detected a glycosylated isoform of the ATP synthase α subunit in bovine heart tissue, in a primate cell line (COS-7) and in the *Caenorhabditis elegans* primary embryonic cell line [99]. These results indicate that glycosylated isoforms of proteins with established mitochondrial function exist and may be more common than previously appreciated. Computational prediction of OSCP (P13621 sequence) glycosylation sites revealed two *O*-glycosylation sites, one of which is near to Helix 6, contacting α^E^ [80] (NetOGlyc 4.0 algorithm [100]), thus suggesting an involvement in the modulation of enzyme activity. Interestingly, in rat cardiac myocytes, an increase of *O*-glycosylation of Complex I, III and IV is associated with impaired respiratory activity [96]. Taken together, these findings open questions about where in the cell and, consequently, by which glycosyltransferases these proteins, including OSCP, become glycosylated and the nature of glycosylation (the attachment site and structure of glycans).

Phosphorylation of OSCP was reported for the first time by Hopper *et al.* [6], who analyzed the phosphoproteome of the mitochondrial matrix in porcine heart, using the phosphorylation targeted dye, ProQ Diamond, in conjunction with 2D gel and mass spectrometry. A phosphorylated form of OSCP has been later found also in porcine heart by the Balaban group, who extensively studied mitochondrial phosphoproteome with PhosTag and ^32^P labeling (for a review of ATP synthase phosphorylation, see [89]). In particular, the OSCP residues, Ser155 and Thr145, have been found phosphorylated in human skeletal muscle and pig heart, respectively [88,101]. Their location near the α_E_ subunit suggests that phosphorylation may participate in the regulation of motor function, but no evidence has been yet reported. Nevertheless, and in spite of these advances, kinases and phosphatases involved in ATP synthase phosphorylation are still poorly defined, and further work will be needed to understand the consequences of phosphorylation on enzyme activity.

The first extensive proteomic study of mitochondrial protein acetylation was performed in mouse liver mitochondria [91] and revealed that more than 20% of mitochondrial proteins contain acetylated lysine residues, including OSCP and the other peripheral stalk subunits, the F_1_ α, β, γ subunits and the F_O_ g and A6L subunits, for which the acetylated sites have been identified. More recently, acetylation of α and OSCP subunits was confirmed by Wei *et al.* [8], who discovered that Sirtuin3, which belongs to a large family of NAD^+^-dependent protein deacetylases, interacts with OSCP and activates the enzyme through deacetylation of α and OSCP subunits. Such a finding is consistent with the ability of acetylation to neutralize the positive charge of lysine residues, as well as to increase the hydrophobicity and size of the lysine side chains, causing potential alterations of the protein propensity to interact with other proteins. Thus, the acetylation of OSCP can induce a conformational change of the protein itself or prevent complex formation with other proteins. The relevance of acetylation was confirmed by the observation that potentially every major metabolic enzyme is acetylated, both inside and outside the mitochondria [102,103].

Interestingly, selective immunoprecipitation of OSCP from mitochondrial lysates revealed that OSCP is ubiquitinated in colon cancer cells (human COLO 205 cell line) together with high levels of the expression of Hsp90 [92]. OSCP ubiquitination was not observed in an MS analysis of mouse heart, where, instead the α, β, γ and b subunits were found to be ubiquitinated [104], suggesting a specific role of OSCP ubiquitination in cancer cells.

In summary, several PTMs have been identified in the OSCP subunit, especially phosphorylation and acetylation, for which specific sites have been found (Table 2). However, for most of them, the functional consequences are still unknown, as well as the enzymes involved in these modifications.

## 5. OSCP Interactors: CyPD and PTP Formation

ATP synthase activity is regulated by the reversible association of a variety of regulatory peptides, including CyPD. Beside the already mentioned IF_1_, an inhibitory interaction with protein kinase Cδ at subunit d of the lateral stalk has been described, whose disruption attenuated the injury resulting from prolonged oxygen deprivation in neonatal cardiac myocytes [105]. Conversely, the Ca^2+^-dependent association of S100A1 with F_1_ improved the enzyme performance in cardiomyocytes [106]. Binding of Bcl-X_L_ to the β subunit and binding of Factor B to the membrane-embedded part of F_O_ increased the aerobic ATP production by blocking a proton leak in healthy neurons [107] and in animal mitochondria [66,108], respectively. In addition, three new interactors have been described that selectively bind OSCP, *i.e.*, the already mentioned Sirtuin3, CyPD and, possibly, p53 [109].

Sirtuins control a variety of cellular function via their NAD^+^-dependent deacetylase activity [110]. Sirtuin3 is the best characterized sirtuin in mitochondria and has emerged as a major regulator of mitochondrial metabolism and energy homeostasis through mitochondrial protein deacetylation [111]. As already mentioned, Wei and co-workers discovered that endogenous Sirt3 interacts with OSCP and mediates the deacetylation of the α and OSCP subunits. Interestingly, these authors found that ATPase acetylation and Sirtuin3 expression are altered in human cells harboring a pathogenic mtDNA mutation (the 4977 bp deletion), as well as under increased ROS production in human cells [8].

p53 is a transcription factor rapidly activated in response to multiple stresses regulating hundreds of genes implicated in cell cycle, senescence, apoptosis, metabolism and DNA repair [112]. For a human cell model expressing mitochondrially-targeted p53 (with a vector in which the sequence encoding a mitochondrial import leader was fused to the 5′ end of wild-type p53), a transcription-independent activity of p53 able to increase oxygen consumption and to decrease ROS production in the absence of acute stress has been recently reported. By immunoprecipitation and mass spectrometry, the authors demonstrated that p53 interacts with OSCP, taking part in the assembly or stabilization of the mature F_O_F_1_ complex, thus suggesting that the mitochondrial fraction of p53, although very low, may be an important regulator of mitochondrial physiology, potentially exerting tumor suppression [109]. However, interaction domains between p53 and Sirt3 with OSCP have been not yet been determined.

CyPD belongs to a ubiquitous protein family with peptidyl-prolyl *cis-trans* isomerase (PPIase) activity, which is inhibited by cyclosporin (Cs) A [113]. CyPs share a common domain of approximately 109 amino acids, the CyP-like domain [17,114]. In spite of this, CyPs do not play a general role in protein folding, and the existence of tissue- and organelle-specific isoforms with the proper targeting and/or retention sequence(s) suggests that each CyP regulates a restricted number of unique partner proteins [114,115].

CyPD is the unique mitochondrial isoform of CyPs in mammals and is involved in the regulation of the PTP, but is not a structural pore component. PTP is a conserved high-conductance channel located in the inner mitochondrial membrane (IMM) that allows the diffusion of solutes up to about 1500 Da [116]. PTP openings of a short duration lead to transient IMM depolarization [117] and may be caused by physiological stimuli [118]; while long-lasting openings cause permanent depolarization, loss of ionic homeostasis, depletion of matrix pyridine nucleotides, resulting in respiratory inhibition, and the generation of reactive oxygen species (ROS). Moreover, the matrix swelling occurs as a consequence of its high oncotic pressure, and the outer mitochondrial membrane (OMM) may disrupt with the release of pro-apoptotic intermembrane proteins, including cytochrome *c*. Thus, long-lasting PTP opening may represent a point of no return in cell commitment to death, which can occur either through apoptosis (if enough ATP is present to sustain caspase activity) or through necrosis (when ATP is depleted) [5].

The role of CyPD as a PTP inducer was suggested by the demonstration that addition of CsA to isolated mitochondria desensitizes the PTP, in that pore opening requires about twice the Ca^2+^ load necessary to open the pore in the absence of CsA [119]. Genetic ablation of the *Ppif* gene (which encodes for CyPD in the mouse) has confirmed that CyPD is the mitochondrial receptor for CsA, and it is responsible for the modulation of the PTP, both *in vitro* and *in vivo* [120–123]. The crystal structure of human CypD in complex with CsA demonstrated that it is composed of eight β-strands, two α-helices and one 3_10_ helix [124].

A major step in the mechanistic understanding of the role of CyPD in PTP regulation has been the discovery that CyPD masks an inhibitory site for Pi, which is the actual PTP desensitizing agent [125]. Furthermore, PTP modulation by CyPD is affected by CyPD phosphorylation [5], acetylation [126] and nitrosylation [127]. Not surprisingly, several regulatory interactions of CyPD have been reported in the literature, including Hsp90 and its related molecule, TRAP-1 [128], Bcl-2 [129], ERK-2/GSK-3 [5] and possibly p53 [130], which also seems to bind OSCP [109].

The identification of CyPD interaction with the OSCP subunit of ATP synthase was an essential step for the identification of the PTP, whose molecular nature was still a matter of conjectures [2]. The previous model still postulated that PTP is formed by a supramolecular complex, including the voltage-dependent anion channel (VDAC) of the OMM, as well as the adenine nucleotide translocator (ANT) and the phosphate carrier (PiC) located in the IMM, but genetic testing has excluded this possibility for all of these proteins [2].

We observed that in mammals, CyPD binds the ATP synthase peripheral stalk in an apparent ratio of 1:1:1:1 with the OSCP, b and d subunits; that this interaction is favored by Pi, which exerts multiple effects on PTP [125], as well as on ATP synthase [131]; and that the interaction is competed by CsA concentrations known to displace CyPD from the IMM [132]. Importantly, CyPD preferentially binds to the ATP synthase dimers, causing a decrease of specific activity that can be reversed by CsA [133]. Consistent with a regulatory interaction, the stimulatory effect of CsA was lost in *Ppif*^−/−^ mitochondria, indicating that it was mediated by CyPD, while the assembly of the ATP synthase was unaffected by CyPD ablation [133].

**Figure 2.**
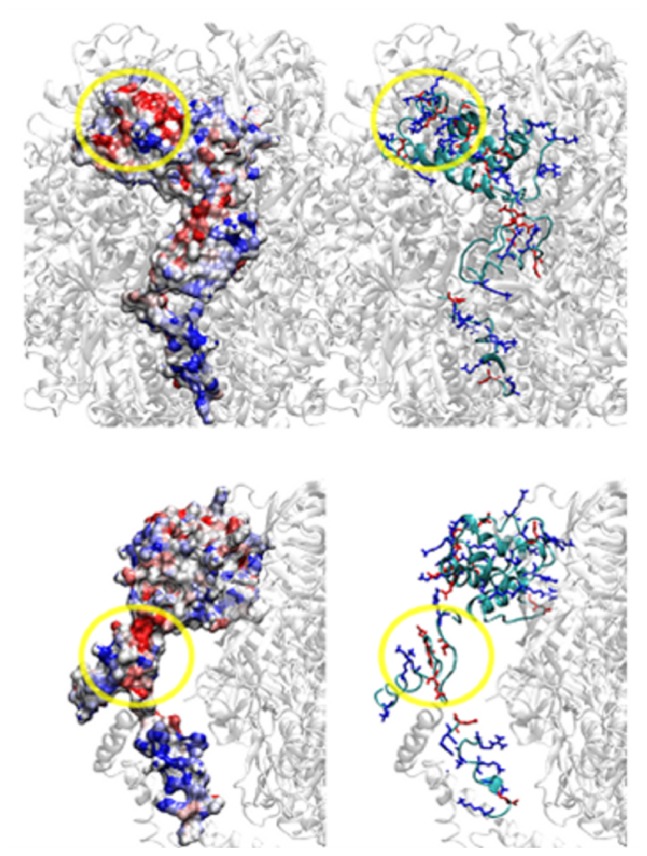
Surface potential regions on OSCP. The two lowest potential regions on OSCP are highlighted by a yellow circle. The average surface atom potential is displayed on the left; the cartoon structure is shown on the right with positively (blue) and negatively (red) charged side chains. The color code for the potential is: saturated red, −8.0 kJ/(mol·q); saturated blue, 8.0 kJ/(mol·q). The F_1_ structure is shown as transparent. The yellow circle in the upper panel is located at OSCP residues E48, D71, E76 and F78. The latter region, discussed in the text, encompasses Helices 3 and 4. For the sake of completeness, the yellow circle in the lower panel is located at the other lowest potential region located at OSCP residues H112, E115, V116, E128 and E133. The surface potential is computed as previously described [4,149].

CyPD interaction with OSCP (which appears to be mostly electrostatic in nature) was confirmed by immunoprecipitation of OSCP from mitochondria [4]. When ATP synthase was immunoprecipitated from mitochondria with decreased OSCP levels, decreased levels of CyPD were detected, as well. A study of surface potentials and isopotential curves of CyPD and OSCP in the ATP synthase complex identified putative binding regions of CyPD on OSCP at the region overlapping with Helices 3 and 4, which represent the lowest (*i.e.*, most negative) average surface potential regions, as shown in Figure 2, where OSCP in the context of ATP synthase is depicted. These helices are also the binding site of benzodiazepine (Bz)-423, a well-characterized inhibitor of ATP synthase (Figure 2) [78]. Treatment of mitochondria with Bz-423 induced CyPD displacement from ATP synthase, suggesting competition for a common binding site. Such localization is therefore consistent with the CyPD inhibitory activity of ATP synthase and may allow CyPD binding to all classes of dimers, including those displaying small angles between two F_1_, which have the peripheral stalks in contact, mutually hiding their subunits, except OSCP. These latter dimers probably form upon IF_1_ binding [3,58], *i.e.*, when ATP synthase hydrolyses ATP generating high Pi, which, in turn, is necessary for CyPD binding [133]. Further work is needed to define the amino acids involved in these interactions.

Consistent with the involvement of ATP synthase in PTP formation, mitochondria treated with Bz-423 or expressing decreased levels of OSCP also decreased the threshold Ca^2+^ required for PTP opening, revealing a striking analogy between the effects of Bz-423 and OSCP on PTP and ATP synthase. Direct evidence that PTP forms from ATP synthase was obtained by electrophysiology, which demonstrated that the addition of Bz-423 in the presence of Ca^2+^ to ATP synthase dimers isolated and incorporated in azolectin bilayers triggered Ca^2+^-dependent currents with features indistinguishable from those of the PTP [134], which were inhibited by Mg^2+^-ADP and by AMP-PNP (γ-imino ATP), a nonhydrolyzable ATP analog. Taken together, these findings prompted us to hypothesize that Ca^2+^, which is able to sustain only uncoupled ATP hydrolysis [29], replaces Mg^2+^ and induces conformational changes in F_O_, which could then mediate PTP formation from ATP synthase dimers. OSCP may thus act as a negative modulator of PTP affecting Ca^2+^ accessibility, except when it binds CyPD or Bz-423, which favor PTP formation.

Beyond CyPD and Ca^2+^, many other effectors regulate the open-closed transitions of PTP, including Mg^2+^, Mn^2+^, nucleotides and low pH (which favor its closure); and low membrane potential, high Pi, and ROS (which favor its opening) [118,135]. ROS may induce the PTP, but also form as a consequence of PTP opening, in a feed-forward loop that may stabilize the pore in the open conformation. The F_O_F_1_ ATP synthase potentially accommodates all these PTP pathophysiological effectors, since divalent cations, nucleotides and Pi bind to the catalytic sites of F_1_, and the membrane potential and pH regulate the catalytic activity, as well as IF_1_ binding; however, the underlying conformational changes responsible for PTP formation are still far from being characterized.

Interestingly, OSCP contains one conserved histidine residue (His 112 in the bovine enzyme) exposed to the matrix and located in one of the lowest surface potential regions (Figure 2), which could be involved in the regulation of PTP by pH and ROS. In fact, previous studies highlighted an essential role of matrix-exposed histidine residue(s) in PTP regulation; histidine protonation at low pH indeed induced CyPD release and PTP closure [132,136]. Moreover, *in vitro* experiments demonstrated that oxidation of critical histidine residue(s) located on the PTP matrix side by singlet oxygen photogenerated *in situ* favored the PTP closed conformation by causing a secondary drop of reactivity to the cross-linking of pore-activating cysteine residues [137]. OSCP also contains a unique cysteine (Cys 118 in bovine mitochondria) conserved in mammals, whose function in ATP synthase catalysis is unclear [69]. We suspect that this residue may be involved in PTP activation by forming a dithiol cross-linking with a cysteine of another matrix protein, possibly only when His 112 is not oxidized. On the other hand, ATP synthase from various organisms is susceptible to different ROS species produced in *in vitro* experiments [138–140], as well as to oxidative/nitrative stress associated with central nervous system (CNS) disorders [141,142], caloric restriction [143] and aging [144,145]. We showed that isolated F_1_ from bovine heart ATP synthase is selectively inactivated by hydrogen peroxide through redox-active iron-protein adducts, probably generating highly reactive oxygen species [139]. More recently, an exclusive target of singlet oxygen and hydrogen peroxide has been identified in a highly conserved methionine-cysteine cluster of the chloroplast γ subunit that is essential for the enzyme coupling and whose oxidation appeared to be directly involved in the loss of enzyme activity [146]. Due to its conservation, it is tempting to hypothesize that also this cluster may be a candidate for ROS-mediated PTP modulation. Other selective ATP synthase targets of ROS may be three of the five Trp residues of the d subunit identified in human heart mitochondria [147] and a single Trp of the α subunit identified in *Podospora anserina* and possibly involved in protein quality control [148]. Their oxidation may occur as a consequence of PTP formation. Moreover, in *Podospora anserina*, an age-related post-translational modification of OSCP has been identified by 2D-PAGE and matrix-assisted laser desorption/ionization-Time-of-Flight mass spectrometry (MALDI-TOF MS) whose nature is still to be clarified [144].

## 6. Concluding Remarks

Through its contacts with the F_1_ α_3_β_3_ hexamer and the peripheral stalk, OSCP not only ensures the structural and functional coupling between F_O_ and F_1_, which is necessary for ATP synthesis, but also modulates the enzyme complex and is the target of inhibitors and interactors, including CyPD. CyPD affects ATP synthase activity and, most importantly, decreases the threshold matrix Ca^2+^ required for PTP opening. This finding, together with the electrophysiological demonstration that ATP synthase dimers give rise to Ca^2+^-dependent currents with features indistinguishable from those of the PTP, indicate that ATP synthase is directly involved in PTP formation and that OSCP plays a fundamental role in this process, acting as a sensor for signal(s) that switch the enzyme of life in a channel able to induce cell death. CyPD and OSCP are the targets of signaling pathways (such as phosphorylation and acetylation) and of common interactors (such as Hsp90). Many open questions remain, in particular: (i) How does CyPD interaction with OSCP change the apparent affinity of the ATP synthase for Ca^2+^, triggering a conformational change that leads to PTP formation? (ii) Where is (are) the Ca^2+^ binding site(s)? (iii) Is the redox sensitivity of the transition of the ATP synthase to PTP formation mediated by a dithiol-disulfide interconversion, and where are the relevant cysteines located? (iv) Is the pH-dependent inhibition of the PTP due to IF_1_ binding, which is pH-dependent? These and many other questions can now be addressed with the powerful methods of genetics, and we have little doubt that the future has a lot in store toward our molecular understanding of the permeability transition.

## Figures and Tables

**Table 1.** Equivalence of subunits of ATP synthase from different sources^1^.

Mitochondria		*E. coli*, *P. modestum* and *A. woodii*	Chloroplast, cyanobacteria 2 and rhodobacteria 3

Bovine	Yeast		
α	α	α	α
β	β	β	β
γ	γ	γ	γ
δ	δ	ɛ	ɛ
ɛ	ɛ	-	-
OSCP	OSCP	δ	δ
b	4 or b	B 4	b and b′ (I and II)
A6L	8	-	-
F6	h	-	-
a	6 or a	a	a (IV)
c	9 or c	c	c (III)
d	d	-	-
e	e	-	-
f	f	-	-
g	g	-	-
-	i/j	-	-
-	k	-	-
MLQ	-	-	-
AGP/DAPIT	-	-	-

1 Subunit equivalence is based on sequence homology;

2 *Synechococcus*;

3 *Rhodospirillum rubrum* and *Rhodopseudomonas blastica*;

4 ATP synthase from *E. coli* and *P. modestum* have two copies of the b subunit and ATP synthase from chloroplasts, cyanobacteria and rhodobacteria have one copy of each b and b′. The b and b′ are homologous; -, no subunit equivalence.

**Table 2.** Post-translational modifications of OSCP.

Modification	Method	Residue	Organism/Tissue	Reference
**Acetylation**	MS 1	K60, K70, K159, K162, K172, K176, K192	Mouse/Liver	[91]
	K Ab		Human 143B osteosarcoma cells	[8]
**Phosphorylation**	ProQ		Pig/Heart	[6]
	PhosTag		Pig/Heart	[87]
	^32^P		Pig/Heart	[90]
	MS	T145	Pig/Heart	[89]
	MS	S155	Human/Muscle	[88]
**Glycosylation**	Leptin resin		Bovine/Heart	[7]
**Ubiquitination**	Ub Ab		Human colon cancer cells	[92]

1 Abbreviations: MS, mass spectrometry; K Ab, anti-lysine antibody; Ub Ab, anti-ubiquitin antibody.
